# Clinicogenomic Landscape and Function of *PIK3CA*, *AKT1*, and *PTEN* Mutations in Breast Cancer

**DOI:** 10.1200/PO-25-00609

**Published:** 2026-04-09

**Authors:** Jacqueline J. Tao, Saumya D. Sisoudiya, Hanna Tukachinsky, Alexa B. Schrock, Ericka M. Ebot, Smruthy Sivakumar, Ethan S. Sokol, Neil Vasan

**Affiliations:** ^1^ Columbia University Irving Medical Center, New York, NY; ^2^ Foundation Medicine Inc, Cambridge, MA; ^3^ Department of Medicine, New York University Grossman School of Medicine, New York, NY; ^4^ Perlmutter Cancer Center, NYU Langone Health, New York, NY

## Abstract

**PURPOSE:**

To comprehensively characterize the clinical and genomic landscapes of *PIK3CA*, *AKT1*, and *PTEN* alterations and examine their functional and therapeutic implications in AKT-driven breast cancer.

**METHODS:**

Comprehensive genomic profiling of 51,767 breast tumors was performed using FoundationOne CDx or FoundationOne. We examined the genomic landscape of *PIK3CA*, *AKT1*, and *PTEN* alterations and their distribution across clinical variables of interest. Prior deep mutational scanning (DMS) data were used to functionally characterize clinical *PTEN* variants. Real-world clinical outcomes were assessed in patients treated with capivasertib plus fulvestrant.

**RESULTS:**

A total of 29,157 variants were identified across the three genes, including pathogenic variants and variants of uncertain significance. The most frequently altered gene was *PIK3CA* (37.4% of cases), followed by *PTEN* (13.5%) and *AKT1* (5.4%). The most common alterations in each gene were *PIK3CA* H1047R (35.6% of *PIK3CA*-altered cases), E545K (19.7%), and E542K (11.7%); *AKT1* E17K (69.7%); and *PTEN* homozygous copy number deletion (37.3%). *PIK3CA* alterations were less prevalent in patients of African genetic ancestry (27.1% vs 38.6% in European genetic ancestry), whereas *AKT1* and *PTEN* alterations were balanced across ancestries. DMS data on missense *PTEN* mutations revealed that 32.5% showed discordant effects on protein stability and phosphatase activity. A subset of patients with rare AKT pathway variants derived meaningful progression-free survival and overall survival benefit from capivasertib.

**CONCLUSION:**

Here, we present the landscape of *PIK3CA*, *AKT1*, and *PTEN* alterations in, to our knowledge, the largest clinical cohort examined to date. The functional complexity of rare *PTEN* variants underscores the need for functional validation by tools such as DMS. Rare AKT pathway variants may predict clinical benefit from AKT inhibitors and warrant further clinical investigation.

## INTRODUCTION

The AKT pathway regulates critical cellular functions including growth, proliferation, survival, and metabolism and is one of the most frequently altered pathways across cancers. *PIK3CA* encodes PI3Kα, which recruits and activates AKT and downstream signaling through mechanistic target of rapamycin complex 1.^1^ The lipid phosphatase PTEN metabolizes PIP_3_, antagonizing the effects of PI3K. Consequently, aberrant AKT pathway activation primarily arises because of activating mutations in *PIK3CA* or *AKT1* and inactivating alterations in *PTEN*.^2^

**CONTEXT**

**Key Objective**
What is the clinicogenomic landscape of *PIK3CA*, *AKT1*, and *PTEN* alterations in breast tumors, and what are their functional and therapeutic implications?
**Knowledge Generated**
Comprehensive genomic profiling of 51,767 breast tumors identified 29,157 variants across the three genes and revealed that *PIK3CA* alterations were less prevalent in patients of African genetic ancestry. Analysis of published deep mutational scanning (DMS) showed that one third of *PTEN* missense variants had discordant effects on protein abundance versus activity. Clinical benefit was observed in a subset of patients with rare variants treated with capivasertib and fulvestrant.
**Relevance**
AKT pathway variants are common in breast tumors and include many rare variants of uncertain significance. Tools such as DMS can clarify the functional implications of rare variants, which may predict clinical benefit from AKT pathway inhibitors. Ancestry-based differences in *PIK3CA* prevalence should be considered in clinical trials.

The AKT pathway is altered in over 40% of estrogen receptor–positive (ER+) breast cancers.^3^ PI3Kα-specific inhibitors including alpelisib and inavolisib are approved for *PIK3CA*-mutated metastatic breast cancer (MBC),^4,5^ but are often associated with significant toxicities including high-grade hyperglycemia. Recently, the AKT inhibitor capivasertib was approved for patients with ER+ MBC with mutations in *PIK3CA*, *AKT1*, and/or *PTEN* after progression on first-line endocrine-based therapy.^6^ In the CAPItello-291 study, capivasertib combined with fulvestrant more than doubled PFS in patients with qualifying genomic alterations^7^ and demonstrated a favorable toxicity profile compared with older generations of PI3K inhibitors. Notably, clinical benefit was limited to patients harboring *PIK3CA*/*AKT1*/*PTEN* alterations, highlighting their importance as biomarkers of sensitivity to capivasertib.

Despite significant progress in genomic characterization of the AKT pathway,^8,9^ prior studies of *PIK3CA/AKT1/PTEN* have had limited sequencing coverage and analyzed smaller genomic data sets. Early analyses evaluated fewer than 2,000 breast tumors using limited gene panel sequencing,^10,11^ whereas more recent studies have examined up to 7,450 breast cancers using next-generation sequencing.^12^

Given the US Food and Drug Administration approval of capivasertib for patients with breast cancer harboring *PIK3CA*, *AKT1*, or *PTEN* alterations as identified by the companion diagnostic FoundationOne CDx, understanding the genomic, functional, and clinical landscapes of this biomarker triad is increasingly critical. Here, we assess the frequencies, distributions, clinical associations, and co-occurrence patterns of *PIK3CA, AKT1,* and *PTEN* variants in the largest clinicogenomic breast cancer data set analyzed to date (N = 51,767) and integrate genomic findings with functional data and real-world clinical outcomes.

## METHODS

### Comprehensive Genomic Profiling of a Breast Cancer Cohort

Comprehensive genomic profiling (CGP) of formalin-fixed, paraffin-embedded tissue sections was performed using the FoundationOne CDx assay or the laboratory-developed test predecessor, FoundationOne, as part of routine clinical care in a Clinical Laboratory Improvement Amendments–certified, College of American Pathologists–accredited laboratory (Foundation Medicine, Inc, Cambridge, MA). This study included 51,767 breast cancer samples profiled between August 2014 and September 2023. Approval for this study, including a waiver of informed consent and Health Insurance Portability and Accountability Act (HIPAA) waiver of authorization, was obtained from the Western Institutional Review Board (protocol No. 20152817).

### Genomic Analysis

CGP was performed on hybrid capture-selected libraries spanning exons of at least 300 cancer-related genes and select introns of genes frequently rearranged in cancer. Processing of the sequence data and identification of different classes of genomic alterations were performed as previously described.^13,14^ The alterations identified included single-nucleotide variants (missense, nonsense, and splice site mutations), indels (in-frame and frameshift mutations), copy number alterations, and rearrangement events. Alterations were classified as either known or likely pathogenic using annotations from various sources, including the COSMIC database, previous scientific literature (such as truncations and deletions in known tumor suppressor genes), or mutations previously characterized as pathogenic; all other alterations were classified as variants of unknown significance (VUS).

### Histopathologic and Clinical Characteristics

Human epidermal growth factor receptor (HER2) status was determined from the HER2 (*ERBB2*) amplification status based on the FoundationOne or FoundationOne CDx assays. ER status was derived from accompanying pathology reports, where available. Clinical data including the biopsy site were obtained from the submitted test requisition form. Genetic ancestry of each patient was predicted using a single-nucleotide polymorphism–based approach, as previously described.^15^

### Co-Occurrence and Mutual Exclusivity Analyses

The data set was interrogated for patterns of co-occurrence and mutual exclusivity of *PIK3CA*, *AKT1*, and *PTEN* alterations with other cancer-associated genes sequenced. A Fisher's exact test was used to identify significantly co-occurring (odds ratio [OR] > 1) and mutually exclusive (OR < 1) patterns using a false discovery rate (FDR)-adjusted *P* value threshold of .05. A Benjamini-Hochberg FDR correction was applied to adjust for multiple comparisons.

### Functional Characterization of Clinical PTEN Alterations

We examined two published deep mutational scanning (DMS) data sets characterizing the effects of single amino acid substitutions on *PTEN* function. Matreyek et al^16,17^ generated abundance scores for 4,547 *PTEN* variants by measuring intracellular abundance in human cells and then assigned an abundance class of low, possibly low, possibly wild-type (WT)–like, or WT-like to each variant. Mighell et al^18^ generated fitness scores for 6,904 *PTEN* variants by assaying lipid phosphatase activity in yeast and then assigned functional categories (truncation-like, hypomorphic, or WT-like) based on fitness score percentiles. We abstracted abundance and fitness scores corresponding to the *PTEN* missense and nonsense variants in our clinical cohort and assigned functional classifications for each variant. Functional designations from the original publications were consolidated into binary categories of pathogenic (abundance class = low or possibly low; fitness category = truncation-like or hypomorphic) and nonpathogenic (abundance class = WT-like or possibly WT-like; fitness category = WT-like or >WT). For each missense *PTEN* variant, functional classifications derived from abundance versus fitness scores were compared.

### Clinical Outcome Analyses

This study used the US-based deidentified Flatiron Health-Foundation Medicine breast cancer Clinico-Genomic Database (FH-FMI CGDB). Clinical data from the Flatiron Health Research Database^19^ are linked to genomic data, derived from FMI's CGP tests FoundationOne CDx and FoundationOne, in the FH-FMI CGDB by deterministic matching, providing a deidentified data set.^13,14,20^ During the study period, the deidentified data originated from approximately 280 cancer clinics (approximately 800 sites of care) and included 12,601 patients with breast cancer who underwent FMI CGP between January 2011 and December 2024. We analyzed 102 patients who had alterations in *PIK3CA*, *AKT1*, and/or *PTEN* and received capivasertib + fulvestrant in any line of therapy (Data Supplement, Fig S1). Of these, 93 patients had a capivasertib-qualifying variant (CAPIm+) and nine patients had other AKT pathway variants (CAPIm–). We assessed real-world progression-free survival (rwPFS), defined as the time from therapy start to the first progression event or death, and real-world overall survival (rwOS) from therapy start. Institutional review board approval was obtained before study conduct and included a waiver of informed consent based on the observational, noninterventional nature of the study (WCG IRB, Protocol No. 420180044).

### Statistical Analyses

Categorical variables were compared between groups using the Fisher's exact test, with an FDR correction for multiple testing. A *P* value of ≤.05 was considered statistically significant. Statistics, computation, and plotting were performed using R 3.6.1 (R Foundation for Statistical Computing, Vienna, Austria).

## RESULTS

### Baseline Cohort Characteristics

This analysis included 51,767 breast tumors. HER2 amplification was assessed in all samples, of which 8.3% was HER2+. ER status was available in 4,219 cases, of which 57.1% was ER+/HER2–, 33.4% was ER–/HER2–, 4.4% was ER–/HER2+, and 5% was ER+/HER2+. The most common ancestries represented were European (72%) and African (14%). Tumor samples were obtained from a metastatic site (47%), local site (33%), lymph node (12%), or unknown site (8%).

### Prevalence and Spectrum of AKT Pathway Alterations

**FIG 1.**
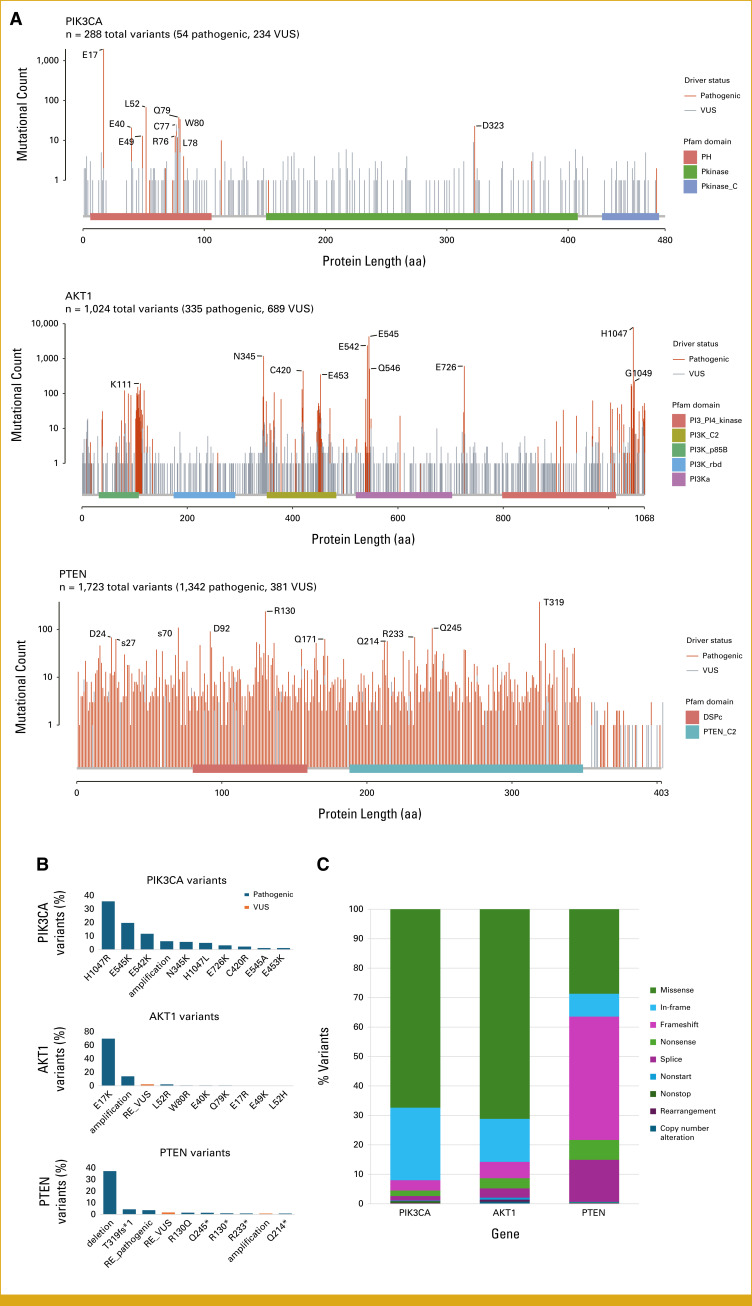
Frequency, distribution, and coding type of *PIK3CA*, *AKT1*, and *PTEN* variants. The most frequently altered AKT pathway gene was *PIK3CA* (37.4% of patients), followed by *PTEN* (13.5%) and *AKT1* (5.4%). (A) Lollipop plots of *PIK3CA*, *AKT1*, and *PTEN* mutations. (B) Prevalence of individual variants in each gene. The most common variants in each gene were *PIK3CA* H1047R (35.6% of *PIK3CA*-altered cases), *AKT1* E17K (69.7%), and *PTEN* deletion (37.3%). (C) Distribution of variant coding types in each gene. The most common variant coding types were *PIK3CA* missense mutation (67.4%), *AKT1* missense mutation (71.2%), and *PTEN* frameshift mutation (41.9%). DSPc, dual-specificity phosphatase catalytic domain; PH, Pleckstrin homology; VUS, variants of unknown significance.

There were 29,157 variants identified across *PIK3CA, AKT1,* and *PTEN*. The most frequently altered gene was *PIK3CA* (37.4% of samples), followed by *PTEN* (13.5%) and *AKT1* (5.4%), including pathogenic alterations and VUS. When restricting to pathogenic alterations only, *PIK3CA* was altered in 36.5% of samples, *PTEN* in 12.7%, and *AKT1* in 4.7%. Among 1,024 distinct *PIK3CA* alterations identified, the most common variant type was missense mutations (67.4% of all *PIK3CA* variants) followed by in-frame mutations (24.6%; Fig 1, Data Supplement, Table S1). The most common mutations were H1047R (35.6% of *PIK3CA*-altered cases), E545K (19.7%), and E542K (11.7%), consistent with known mutational hotspots.^21^ Among 288 distinct *AKT1* alterations identified, 71.2% were missense mutations, predominantly the hotspot mutation E17K (69.7% of *AKT1*-altered cases; Fig 1, Data Supplement, Table S2). Finally, among 1,723 distinct *PTEN* alterations identified, common variant types were distributed between frameshift mutations (41.9% of *PTEN* variants), missense mutations (28.7%), and splice site mutations (14.3%). The most common individual *PTEN* alteration was homozygous loss (37.3% of *PTEN*-altered cases; Fig 1, Data Supplement, Table S3).

Multiple pathogenic *PIK3CA* alterations were seen in 3,148 samples (16.2% of all *PIK3CA*-altered samples and 6.1% of overall cohort). Pathogenic coalterations across genes were most common with *PIK3CA* and *PTEN* (4% of cohort) followed by *PIK3CA* and *AKT1* (0.6%) and then *AKT1* and *PTEN* (0.14%).

### Clinical and Demographic Associations

**FIG 2.**
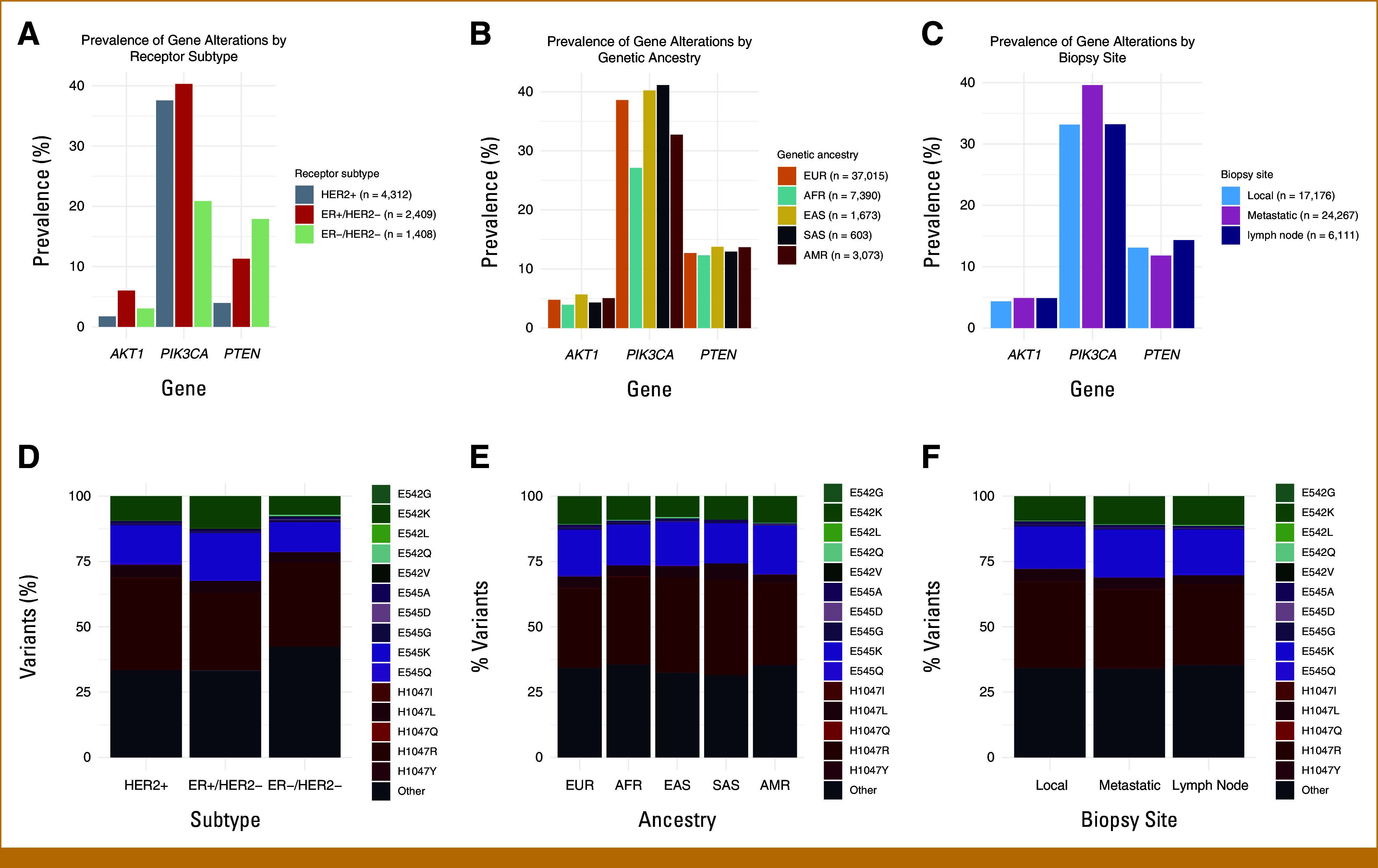
Prevalence of gene variants by breast cancer receptor subtype, genetic ancestry, and biopsy site. (A) *PIK3CA* variants were enriched in ER+/HER2– and HER2+ disease (altered in 40.3% and 37.6% of cases, respectively) and *AKT1* variants were enriched in ER+/HER2– disease (6.0%). By contrast, *PTEN* variants were most prevalent in ER–/HER2– disease (17.9%). (B and C) *PIK3CA* alterations were less prevalent in patients with African ancestry (27.1% *v* 38.6% in European ancestry) and enriched in metastases (39.6% *v* 33.2% in local tumors). (D) *PIK3CA* variants outside the 542, 545, and 1,047 codons were more common in ER–/HER2– disease (42%) compared with ER+/HER2– or HER2+ disease (33% and 33%). (E and F) *PIK3CA* variants by ancestry and biopsy site. ER, estrogen receptor; HER2, human epidermal growth factor receptor 2.

We next examined mutational patterns by receptor subtype, patient ancestry, and biopsy site (Figs 2A-2F, Supplementary Tables S4-S6). *PIK3CA* pathogenic variants were more prevalent in ER+/HER2– and HER2+ disease (40.3%, *P* = 6.0 × 10^−36^ and 37.6% of cases, *P* = 2.3 × 10^−32^) than ER–/HER2– disease (20.9%), consistent with prior reports.^10,22^ However, variants outside the three mutational hotspots were more common in ER–/HER2– (42%) than ER+/HER2– or HER2+ cases (33%, *P* = .002 and 33%, *P* = .002). *AKT1* mutations were most prevalent in ER+/HER2– disease (6.0%) and less common in HER2+ or ER–/HER2– disease (1.7%, *P* = 4.7 × 10^−20^ and 3.0%, *P* = 3.5 × 10^−5^). By contrast, *PTEN* alterations were enriched in ER–/HER2– (17.9%) compared with ER+/HER2– and HER2+ disease (11.3%, *P* = 1.8 × 10^−8^ and 3.9%, *P* = 2 × 10^−57^).

*PIK3CA* pathogenic alterations were less prevalent in patients with African ancestry, both in the full cohort (27.1% *v* 38.6% in Europeans, *P* = 9.6 × 10^−81^) and in the ER+/HER2– subgroup (31.2% *v* 41.1% in Europeans, *P* = .002). In addition, among ER+/HER2– cases, patients with South Asian ancestry had a higher prevalence of *PIK3CA* pathogenic alterations (68.9% *v* 41.1% in Europeans, *P* = .002). *PIK3CA* pathogenic variants were enriched in metastases compared with local tumors (39.6% *v* 33.2%, *P* = 6 × 10^−41^). The distribution of individual *PIK3CA* variants was similar across ancestries and biopsy sites. The prevalence of pathogenic *AKT1* and *PTEN* alterations was similar across all ancestries and biopsy sites.

### Co-Occurrence and Mutual Exclusivity Analyses

**FIG 3.**
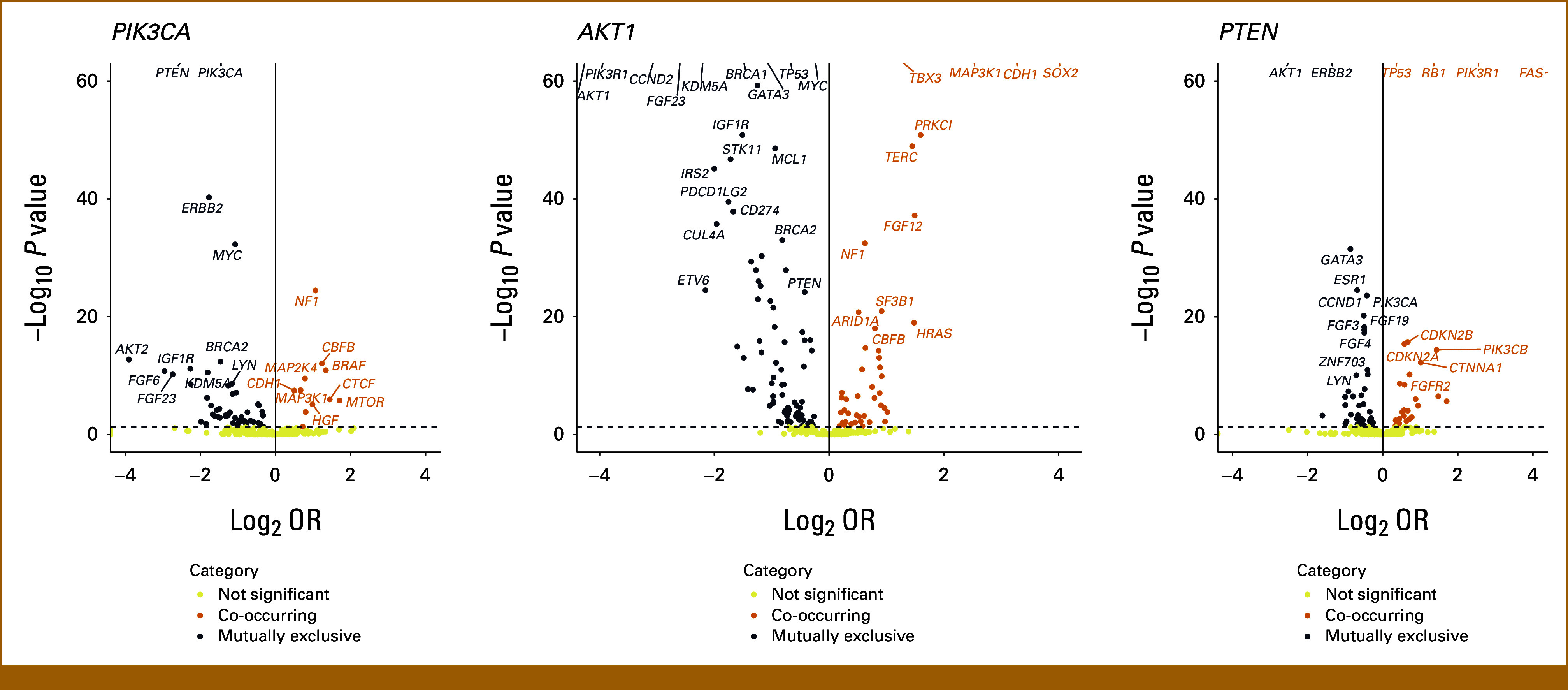
Co-occurrence and mutual exclusivity analyses of pathogenic variants in *PIK3CA*, *AKT1*, and *PTEN*. Pathogenic variants in these three genes were all mutually exclusive to each other. Notable co-occurrences included *PIK3CA* with *SOX2, PRKCI, MAP3K1, TERC, CDH1,* and *TBX3*; *AKT1* with *NF1* and *CDH1*; and *PTEN* with *FAS*, *TP53*, and *RB1*. Notable mutual exclusivities include *PIK3CA* with *TP53, BRCA1/2,* and *STK11*; *AKT1* with *ERBB2* and *MYC*; and *PTEN* with *ERBB2, GATA3,* and *ESR1*. Y-axis is capped at a *P* value of 10E-60.

Next, we characterized patterns of co-occurrence between pathogenic variants in *PIK3CA*, *AKT1*, and *PTEN* and other sequenced genes (Fig 3, Data Supplement, Tables S7-S9). *PIK3CA*, *AKT1*, and *PTEN* pathogenic alterations were all strongly mutually exclusive with each other (*PIK3CA/AKT1* OR, 0.25, *P* < 10^−60^; *PIK3CA/PTEN* OR, 0.74, *P* = 6.6 × 10^−25^; *AKT1/PTEN* OR, 0.20, *P* < 10^−60^), suggesting that a single driver mutation may be sufficient for pathway activation. Notably, the degree of mutual exclusivity was lowest between *PIK3CA* and *PTEN*, reflecting their higher rates of comutation compared with the other gene combinations.

*PIK3CA* was significantly coaltered with *SOX2, PRKCI, MAP3K1, TERC, CDH1,* and *TBX3*. Co-occurrence with *SOX2* is not well-described in the breast cancer literature although there are reports suggesting a role of *PIK3CA* and *SOX2* coamplification in the pathogenesis of head and neck cancers.^23^ There are well-known associations of *PIK3CA* with *CDH1* and *TBX3*, whose losses are hallmarks of invasive lobular carcinoma (ILC),^24,25^ and *MAP3K1*, which is associated with the luminal A phenotype.^26-28^ These findings are consistent with the enrichment of ILC and luminal A breast cancers for *PIK3CA* mutations. Several tumor suppressors were mutually exclusive with *PIK3CA* including *TP53, BRCA1/2,* and *STK11*. Other notable mutual exclusivities included *PIK3R1, FGF23, CCND2, MYC, KDM5A,* and *GATA3*.

*AKT1* was significantly coaltered with *NF1* and *CDH1*, an association previously described in ILC.^29^ There were several shared co-occurrence patterns with *PIK3CA*, including *MAP3K1*, which may reflect the increased prevalence of both *PIK3CA* and *AKT1* in ER+/HER2– tumors*. AKT1* was mutually exclusive with *ERBB2* and *MYC*.

*PTEN* was significantly coaltered with *FAS* (both located on 10q23) and the tumor suppressors *TP53* and *RB1*. *PTEN* is known to be an essential mediator of Fas-mediated apoptosis, and *PTEN* loss can lead to impaired apoptosis and increased cell survival.^30^ Combined *PTEN* and *TP53* loss is known to be associated with poor therapeutic response and prognosis, particularly in triple-negative breast cancers.^31,32^ Notable mutually exclusivities included *ERBB2, GATA3,* and *ESR1*.

Co-occurrence analyses of VUS in the three genes of interest showed weakly significant results (Data Supplement, Fig S2 and Tables S7-S9).

### Functional Characterization of Clinical PTEN Alterations

Among the >1,700 clinical *PTEN* variants identified in our cohort, a substantial proportion are VUS. To explore the functional implications of these clinical *PTEN* variants, we examined two published DMS data sets characterizing the effects of single amino acid substitutions on *PTEN* abundance (protein expression) and fitness (lipid phosphatase activity).^16-18^ Of the 610 single amino acid substitution *PTEN* variants (494 missense, 116 nonsense) in our clinical cohort, 339 (55.6%) had an available abundance score and 536 (87.9%) had an available fitness score; 298 (48.9%) variants had both abundance and fitness scores available (Data Supplement, Table S10).

**FIG 4.**
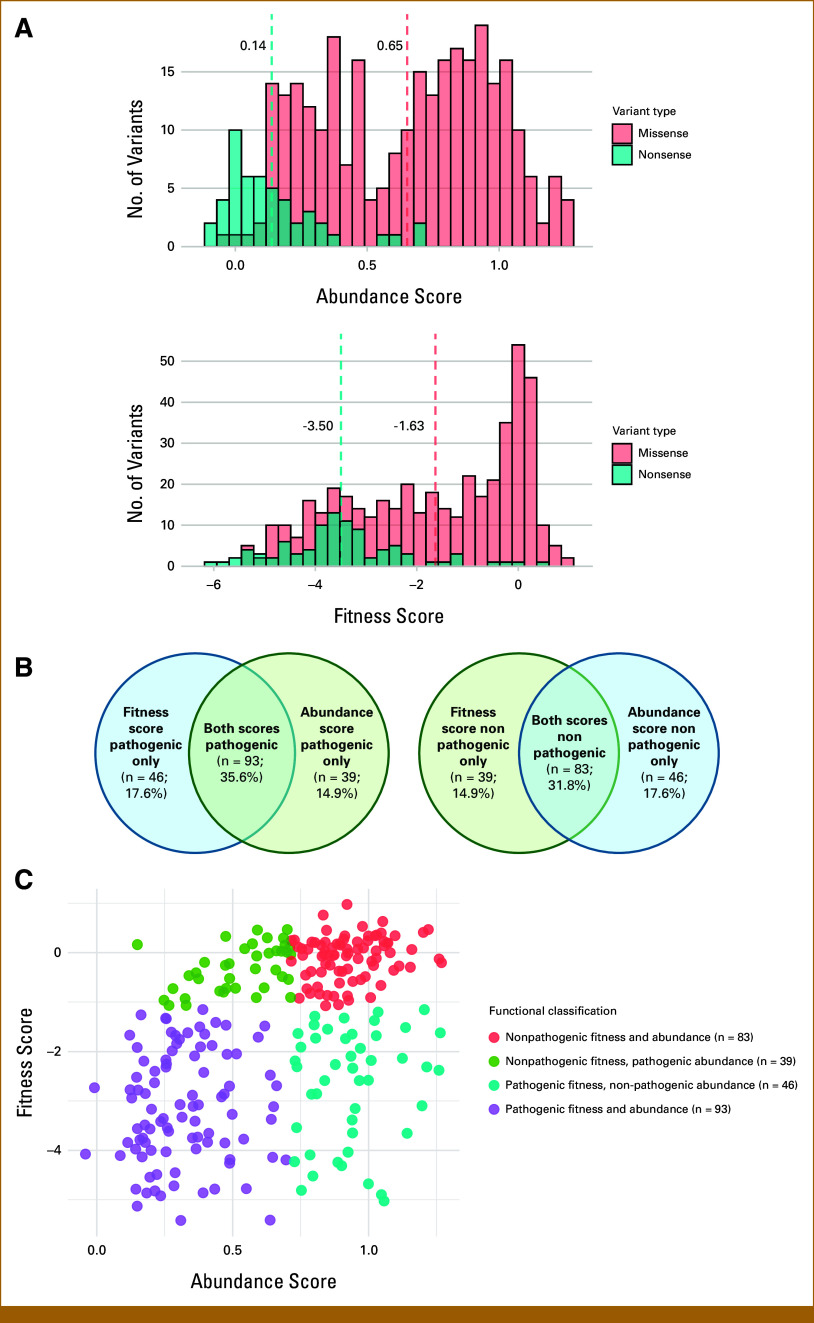
Deep mutational scanning data for missense and nonsense *PTEN* variants. (A) Distribution of abundance and fitness scores among missense versus nonsense variants in the cohort, from the studies by Matreyek et al^17^ and Mighell et al.^18^ Mean abundance and fitness scores for nonsense variants are significantly lower than those for missense variants (mean scores for each group are represented by dashed lines; *P* = 1.12 × 10^−28^ and *P* = 4.93 × 10^−25^, respectively). (B) Set diagrams depicting degree of concordance in functional classification by abundance versus fitness score, for missense variants only. (C) Scatterplot of abundance and fitness scores for missense variants only, colored by concordance in functional classification between the two assays.

Nonsense variants exhibited lower abundance and fitness scores than missense variants (Fig 4A) and were almost all appropriately classified as pathogenic by both abundance score (100%) and fitness score (95.7%). By contrast, there was significant variation in the functional classifications assigned to missense variants. By abundance score, 148 (51.0% of missense variants with available abundance score) were pathogenic and 142 (49.0%) were nonpathogenic; by fitness score, 231 (52.0%) were pathogenic and 213 (48.0%) were nonpathogenic. For missense variants with both abundance and fitness scores available (n = 261; Figs 4B and 4C), 35.6% was classified as pathogenic by both assays, whereas 31.8% was classified as nonpathogenic by both assays, highlighting that a substantial proportion of missense *PTEN* variants do not contribute to pathogenicity. The remaining one third of missense variants were discordantly classified (32.5%), exhibiting either loss of abundance with preserved phosphatase activity (14.9%) or loss of phosphatase activity with preserved abundance (17.6%). These discordances likely reflect the distinct phenotypic effects of mutations in different amino acid residues.

### Clinical Outcomes Among Patients Treated With Capivasertib and Fulvestrant

**FIG 5.**
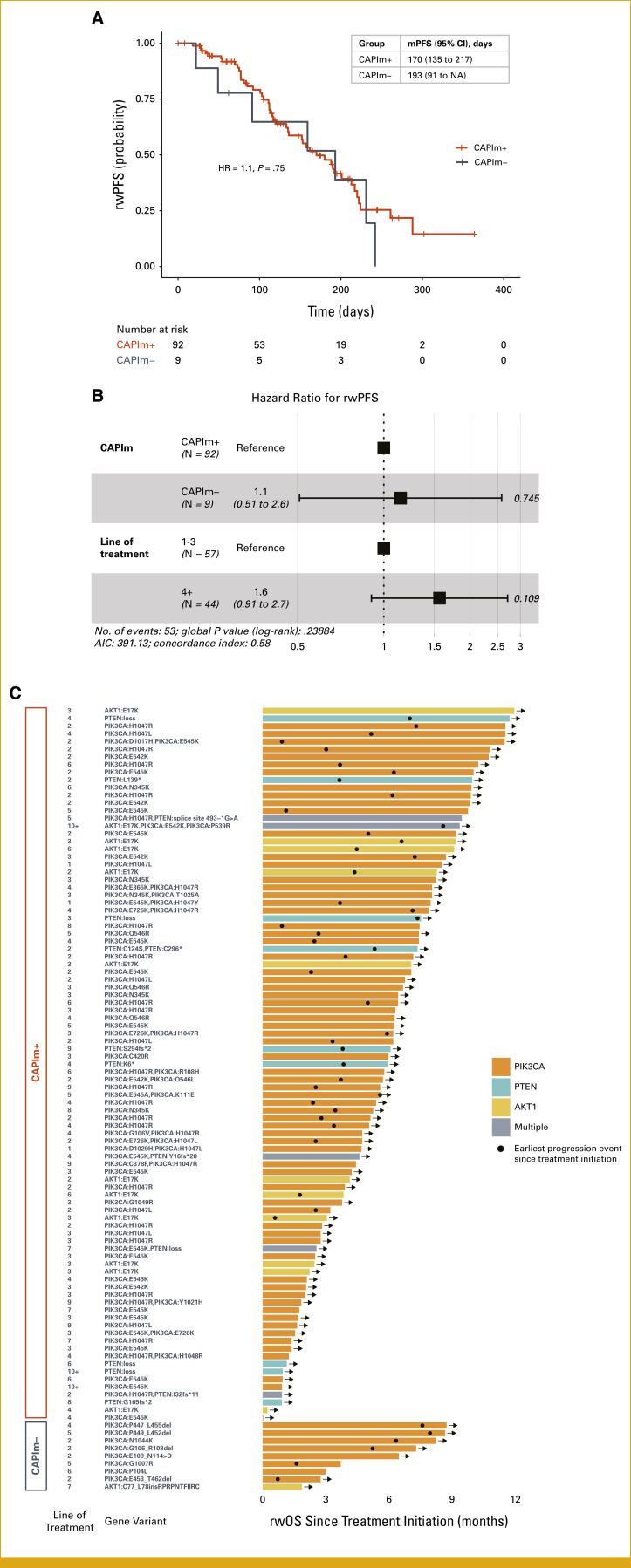
Clinical outcomes among patients treated with capivasertib and fulvestrant. (A) Kaplan-Meier analysis and (B) hazard ratios for multivariate analysis of rwPFS among patients with capivasertib-qualifying variants (CAPIm+) versus other AKT pathway variants (CAPIm–). rwPFS was defined as the time from therapy start to the first progression event or death. Progression events within 14 days of therapy start date were excluded. Patients with no progression event within the line of therapy were censored at the last structured activity date within the line of therapy. Median rwPFS did not differ significantly between groups. A trend toward improved rwPFS was observed in patients treated in earlier (1-3) compared with later (4+) lines of therapy. (C) Swimmer plot showing rwOS from treatment initiation for each patient in the CAPIm+ (top) and CAPIm– (bottom) groups, with annotations indicating line of therapy and genomic profile. rwOS bars are colored by gene variant; black dots indicate earliest progression event after treatment initiation. AIC, Akaike information correction; HR, hazard ratio; LoT, line of treatment; mPFS, median progression-free survival; rwOS, real-world overall survival; rwPFS, real-world progression-free survival.

Finally, we evaluated real-world treatment outcomes among patients receiving capivasertib plus fulvestrant who had capivasertib-qualifying variants (CAPIm+, n = 93) versus other AKT pathway variants (CAPIm–, n = 9). Clinical characteristics did not differ significantly between the groups (Data Supplement, Table S11). Real-world PFS was similar between the groups (median rwPFS 170 day *v* 193 days, hazard ratio, 1.1 [95% CI, 0.51 to 2.6]; *P* = .75; Figs 5A and 5B), demonstrating that meaningful responses can be achieved even in patients with rare AKT pathway variants. Individual rwOS, time of first progression after treatment initiation, treatment line, and gene variant profiles are presented in a swimmer plot (Fig 5C). Clinical benefit was observed in a substantial proportion of patients belonging to both CAPIm+ and CAPIm– groups.

## DISCUSSION

Here we report the largest landscape study, to our knowledge, of *PIK3CA*, *AKT1,* and *PTEN* genomic alterations in breast tumors, encompassing 29,157 alterations across 51,767 samples. The frequency and distribution of *PIK3CA* and *AKT1* alterations were consistent with prior reports.^10-12,22,33-35^ *PTEN* alterations were more common (13.5%) than previously described (≤10%),^11,12^ potentially reflecting broader sequencing coverage.

The most common *PIK3CA* variants observed were H1047R, E545K, E542K, N345K, and H1047L, consistent with previous reports.^34,35^ The roles of nonhotspot mutations remain variably understood although some less common variants including N345K^36,37^ have been associated with alpelisib sensitivity in preclinical studies. We also found that 16.2% of *PIK3CA*-altered samples had multiple *PIK3CA* alterations, similar to previous studies,^38^ which have been shown to result in increased PI3K activity and increased sensitivity to PI3Kα inhibitors. *AKT1* E17K accounted for over two thirds of *AKT1* variants, leaving a notable minority of nonhotspot alterations that remain poorly characterized. A subset of non-E17K variants have been shown to sensitize tumors to AKT inhibition or confer selective hypersensitivity to ATP-competitive AKT inhibitors,^39^ underscoring the need to further evaluate mutant-specific therapeutic vulnerabilities. ER–/HER2– cases had a higher proportion of nonhotspot *PIK3CA* variants than other receptor subtypes; however, given historically negative trials of AKT pathway inhibition in ER–/HER2– breast cancer,^40-43^ the therapeutic implications of this trend remain unclear.

Our ancestry analyses revealed that *PIK3CA* alterations were less common in patients with African ancestry, consistent with smaller prior studies.^44-47^ Interestingly, no such differences were observed in *AKT1* and *PTEN*, suggesting that ancestry-based differences in *PIK3CA* prevalence are gene-specific rather than affecting the AKT pathway globally. This could reflect differential tumor biology that limits the early clonal acquisition of *PIK3CA* mutations in African patients, highlighting one potential biological explanation for lower representation of this population in *PIK3CA* biomarker-based trials. Conversely, *PIK3CA* prevalence was higher in patients with South Asian ancestry and ER+/HER2– disease, underscoring the need for representative patient enrollment in biomarker-based clinical trials.

Our gene association analyses corroborated prior findings of strong mutual exclusivity among pathogenic *PIK3CA*, *AKT1*, and *PTEN* alterations,^10,28,35^ likely reflecting redundancies in AKT pathway activation. Co-occurrence patterns involving *PIK3CA* and *AKT1* were consistent with their increased prevalence in ER+ disease and ILC. We identified a novel co-occurrence between *PIK3CA* and *SOX2*, a combination previously implicated in the pathogenesis of head and neck cancers but not well-described in breast cancers. *PTEN*-altered samples exhibited known enrichment for mutations in other tumor suppressors including *FAS* and *TP53*.

The functional characterization of *PTEN* VUS remains an important challenge in clinical genomics. By leveraging large-scale DMS data sets, we show that nearly one third of missense *PTEN* variants were consistently classified as nonpathogenic by both abundance and fitness-based assays, highlighting the value of functional data in refining clinical variant interpretation. However, discordant classifications in nearly one third of missense variants suggest that a substantial proportion of mutations may not predict straightforward loss of function, warranting further mechanistic investigation as to whether they predict for AKT inhibitor sensitivity. Expanding and refining such data sets will be critical for improving precision in molecular diagnostics and therapeutic guidance.

Finally, in our real-world clinical cohort treated with capivasertib and fulvestrant, patients with rare AKT pathway variants achieved clinically meaningful benefit comparable with that seen in patients with capivasertib-qualifying gene variants. Although sample sizes were small given the recent approval of capivasertib, these findings suggest that therapeutic benefit from AKT inhibition may extend to patients beyond those who meet current biomarker eligibility criteria.

Our study has limitations including the lack of available ER status on a subset of patients although the mutational distributions observed across receptor subtypes were consistent with prior reports. Samples are restricted to US-based patients, which could limit the generalizability of the ancestry-related findings.

In conclusion, this large CGP data set of >51,000 breast tumors reveals a wide spectrum of alterations in *PIK3CA/AKT1/PTEN,* encompassing well-established pathogenic variants and rare variants of uncertain significance. Functional tools such as DMS and continued clinical studies are needed to clarify the roles of lesser-known variants as predictive biomarkers, and ancestry-based differences in *PIK3CA* prevalence should be considered in clinical trials of AKT inhibition.

## Data Availability

A data sharing statement provided by the authors is available with this article at DOI https://doi.org/10.1200/PO-25-00609. All relevant data are provided within the article and its accompanying Supplementary Data. Due to HIPAA requirements, we are not authorized to share individualized patient genomic data, which contain potentially identifying or sensitive patient information. Foundation Medicine is committed to collaborative data analysis, with well-established and widely utilized mechanisms by which investigators can query our core genomic database of >700,000 deidentified sequenced cancers to obtain aggregated data sets. For more information and mechanisms of access, please contact the corresponding author(s) or the Foundation Medicine, Inc Data Governance Council at data.governance.council@foundationmedicine.com. For the data analysis of the clinicogenomic database (CGDB), the data were originated by and are the property of Flatiron Health, Inc and Foundation Medicine, Inc Requests for data sharing by license or by permission for the specific purpose of replicating results in this manuscript can be submitted to PublicationsDataAccess@flatiron.com and cgdb-fmi@flatiron.com.
